# Scrotal and Penile Erythrodysesthesia Associated with Neoadjuvant Capecitabine Chemoradiation

**DOI:** 10.7759/cureus.7724

**Published:** 2020-04-18

**Authors:** Julie Dricken, Erica Pettke, John A Griffin, Henry Y Li, Vivek Mehta

**Affiliations:** 1 Colon and Rectal Clinic, Swedish Cancer Institute, Seattle, USA; 2 Department of Oncology and Hematology, Polyclinic, Seattle, USA; 3 Department of Radiation Oncology, Swedish Cancer Institute, Seattle, USA

**Keywords:** capecitabine, scrotal erythrodysesthesia, neoadjuvant chemoradiation

## Abstract

Capecitabine, a prodrug of fluorouracil, is a component of many chemotherapy regimens used to treat a wide variety of malignancies. One of the most common adverse reactions experienced by those who have been exposed to capecitabine is palmar-plantar erythrodysesthesia (PPE). PPE is a cutaneous manifestation of chemotherapy-related drug toxicity that has signs and symptoms of erythema, edema, pain, ulceration, or desquamation of the palms of the hands and soles of the feet. The signs and symptoms occur with varying severity. There are few reports of the genitalia being similarly affected. The following case describes a patient with locally advanced rectal cancer who experienced erythrodysesthesia secondary to a capecitabine-containing neoadjuvant chemoradiation regimen that primarily and most significantly involved the genitalia.

## Introduction

Palmar-plantar erythrodysesthesia (PPE), also known as hand-foot syndrome, acral erythema, or Burgdorf’s reaction, is a cutaneous side effect associated with some cytotoxic agents used to treat many of the most common malignancies. Some of the available literature makes a distinction between PPE and hand-foot syndrome, with some using the terms interchangeably. For the purposes of this paper, the two terms will be considered to be synonymous. The syndrome is associated with prolonged administration of the systemic agent and appears to be dose-dependent. The onset and severity of the disease can be affected by peak plasma concentrations, total cumulative dose, and administration schedule. It was first described in 1984 when an association with protracted infusion of 5-fluorouracil (5-FU) was noted, but there was no association with bolus 5-FU [1].

Although the present case involves a capecitabine-containing regimen, there are reported associations of PPE with all of the following: doxorubicin (particularly pegylated liposomal doxorubicin), cytarabine (particularly in combination with an anthracycline for treatment of acute leukemia), docetaxel, 5-FU, bleomycin, cisplatin, cyclophosphamide, daunorubicin, doxifluridine, etoposide, fludarabine, gemcitabine, hydroxyurea, idarubicin, ixabepilone, methotrexate, mitotane, paclitaxel, tegafur, thiotepa, vinorelbine. 

Possibly more significant than the agent itself is the drug formulation and administration schedule. The fact that the pegylated liposomal doxorubicin, liposomal daunorubicin, infusional 5-FU, and capecitabine have a stronger association with toxicities, of which PPE is included, than other drug formulations and administration schedules that do not result in such a sustained and prolonged tissue exposure would support this [2].

Various treatments for PPE have been proposed, all with varying and usually disappointing success. The most common and most successful intervention leading to the resolution of PPE is an interruption of therapy [3].

## Case presentation

**Figure 1:**
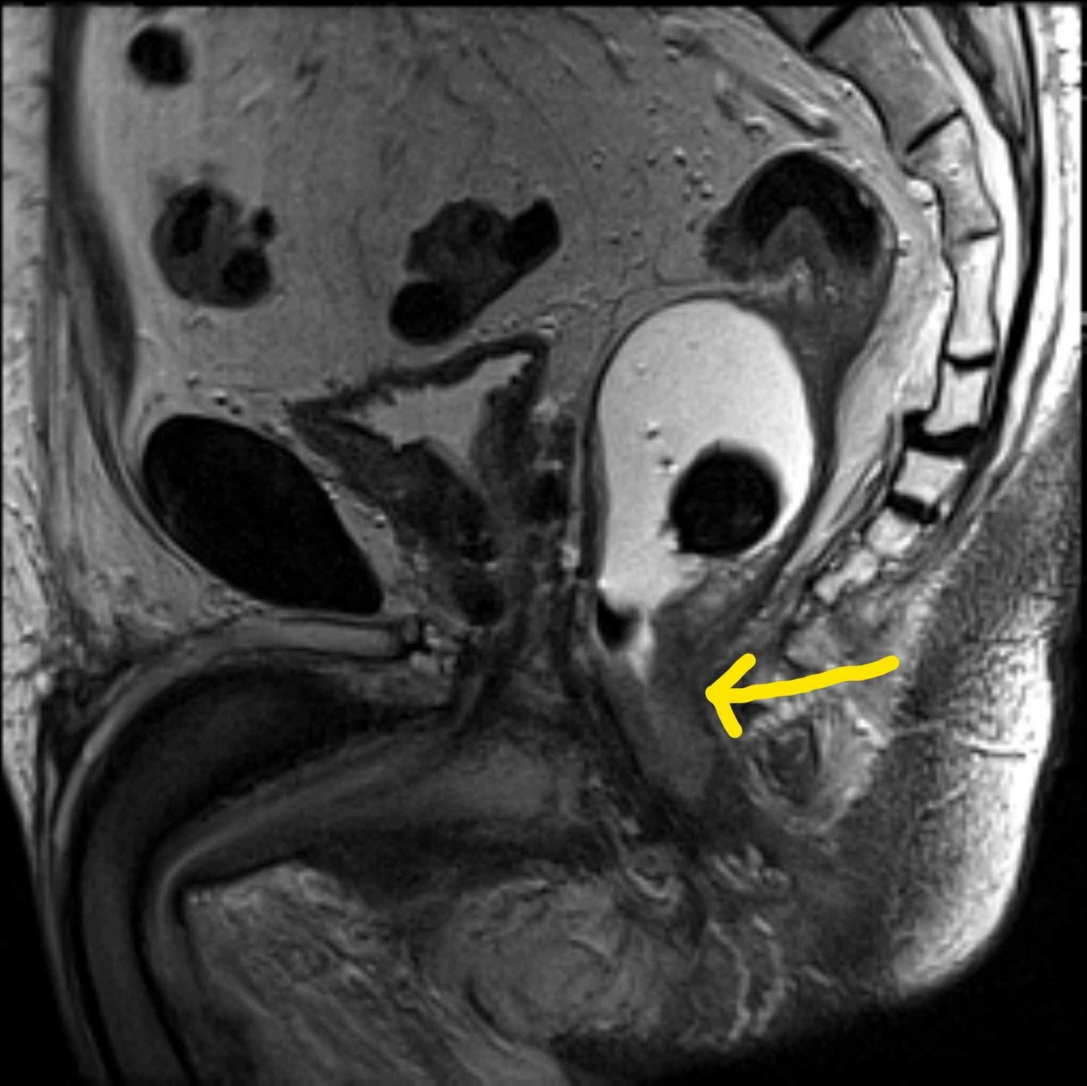
Pelvic MRI showing rectal mass

An 87-year-old man presented with a history of intermittent bleeding per rectum for the last several months. He underwent a colonoscopy and was diagnosed with a moderately differentiated rectal adenocarcinoma with intact mismatch repair protein expression based on biopsy results. Magnetic resonance imaging revealed a T3N0 rectal cancer with extensive involvement of the sphincters, extending from the anorectal junction to 1 cm from the anal verge (Figure 1). Computed tomography images found no evidence of metastatic disease and the carcinoembryonic antigen level was 1.9.

He was described as youthful and had relatively few comorbidities. He had a history of prostate cancer, for which he underwent brachytherapy eight years prior. Genetic polymorphism testing was not done.

The plan for neoadjuvant chemoradiation therapy consisted of capecitabine 825 mg/m^2^ twice daily (1500 mg twice daily) Monday through Friday, skipping non-radiation days, for six weeks; and 5040 cGy of radiation delivered over 28 fractions.

After receiving 11 fractions of radiation therapy, the patient started to experience left groin pain that resolved without intervention. Three days later, he developed numbness of the hands and feet without erythema nor skin changes. Four days later, he again complained of scrotal discomfort. The dose of capecitabine was decreased to 1000 mg every AM and 1500 mg every PM, skipping non-radiation days, for the remaining weeks to complete a total course of six weeks. The patient stopped taking capecitabine, and the decision was made to stop radiation as well. At that point, he had received three weeks of capecitabine, and 14 of 28 fractions (2520 cGy of 5040 cGy) radiation.

**Figure 2:**
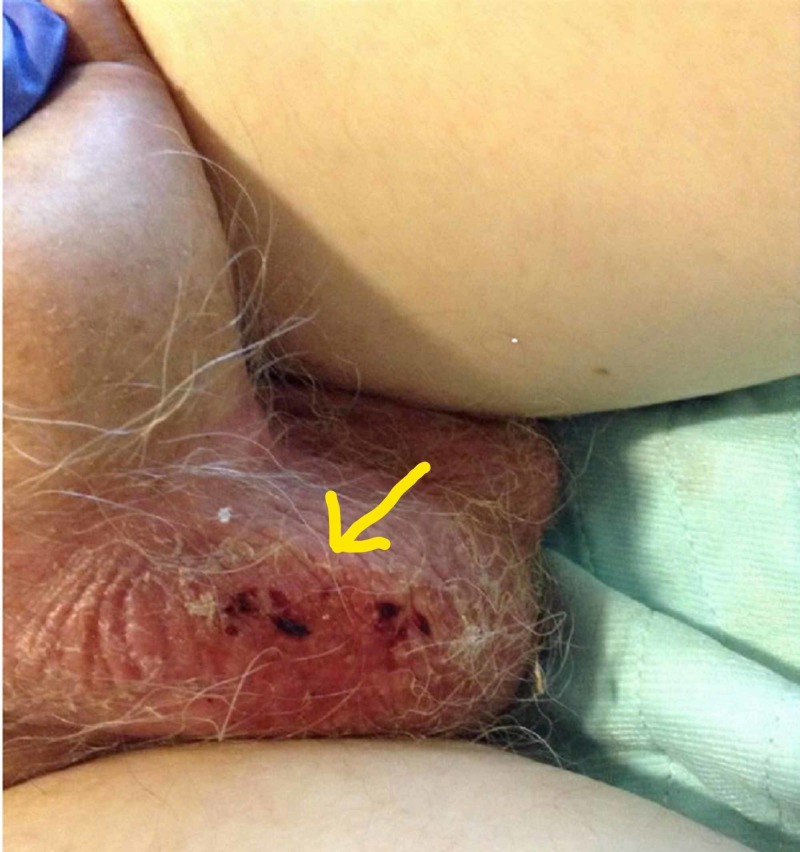
Scrotal erythema and desquamation

**Figure 3:**
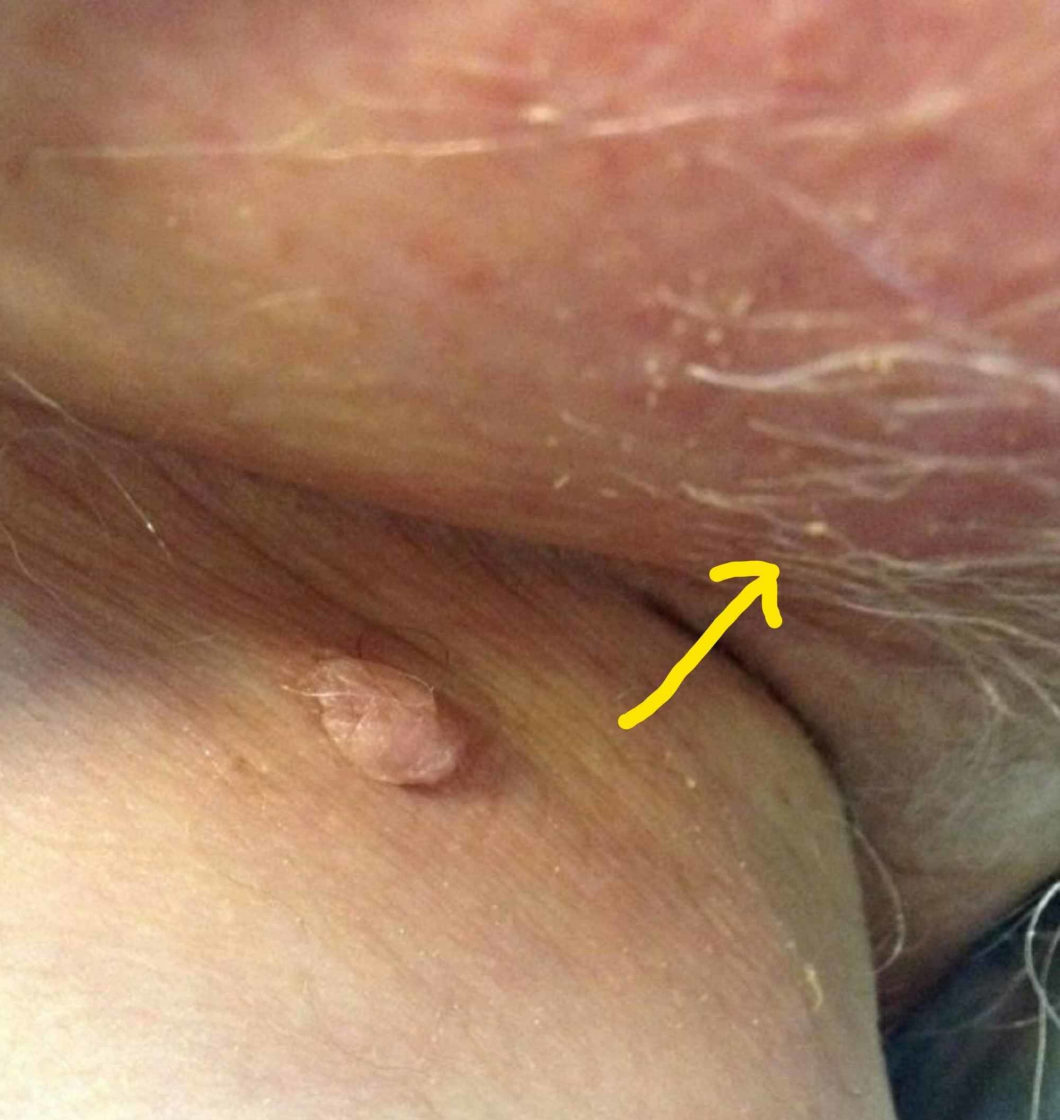
Inguinal and scrotal erythema

One day later, he started to have significant diarrhea and hematuria. Over the next few days, he became dehydrated due to persistent diarrhea and was admitted to the hospital for treatment of dehydration and electrolyte derangements. Physical examination revealed severe scrotal and penile desquamation (Figures 2 and 3). He was seen and evaluated by the wound care team, and empiric treatment was started with silver sulfadiazine cream and antifungal cream. No skin nor wound cultures were obtained. The length of stay was nine days. By 30 days after discharge from the hospital, the pain had completely resolved, erythema nearly resolved, and desquamation significantly improved. 

In order to evaluate the possibility of radiation-induced dermatitis, the radiation plan and delivery data were reviewed. The radiation fields were designed to intentionally avoid and limit the radiation dose to the patient’s genitalia and inguinal region. The initial plan had documented verification by a medical dosimetrist and a medical physicist. On the first day of treatment, a radiation output measurement was performed for each beam to further ensure that the beams were being delivered safely and accurately. These diode measurements were also all within 1.5% of the expected value and normal tolerances. Upon further investigation, no errors in the planning or delivery were identified. The patient's alignment, based on cone-beam CT data, was reviewed and found to have a close agreement between the expected and the acquired image sets. Finally, the alignment data for the C-RAD Catalyst™ system, the system that captures a 3D surface map of the patient, was reviewed and showed accurate alignment. 

On follow up with his colorectal surgeon, the rectal mass was noted to be nodular, mobile, less bulky, and to involve the anal sphincters on the digital rectal exam. Re-staging MRI showed the rectal mass was no longer visible and only fibrosis was noted at its previous location. A flexible sigmoidoscopy demonstrated an ulcerated mass. The mass was biopsied and pathology resulted as moderately differentiated adenocarcinoma. After extensive multi-disciplinary discussions and obtaining a second opinion, he elected to forego further chemoradiation, and also decided against surgery.

## Discussion

PPE is a common side effect of capecitabine with a reported incidence of between 28-58% [4]. As of the time of this case report, there have been eight reported cases of PPE with the involvement of the scrotum and genitalia, including this one [5-8]. Despite the fact that PPE is seen in association with many other drugs used to treat malignancies, it appears as though the involvement of the genitals has been reported more commonly to the administration of capecitabine. Erythrodysesthesia may be seen in other areas of the body that experience increased pressure or temperature, such as the buttocks, under the breasts, in the axillae, and labial area.

Capecitabine, a prodrug of fluorouracil, is a component of many chemotherapy regimens used to treat a wide variety of malignancies. It may be found to be more appealing than 5-FU because it is administered orally. Once swallowed, it is readily absorbed from the gastrointestinal tract. In the liver, a carboxylesterase hydrolyzes much of the compound to 5’-deoxy-5-fluorocytidine (5’-DFCR). Cytidine deaminase, an enzyme found in most tissues, including tumors, then converts the 5’-DFCR to 5’-deoxy-5-fluorouridine (5’-DFUR). Subsequently, the enzyme thymidine phosphorylase hydrolyzes the 5’-DFUR to the active drug 5-FU.

Fluorouracil is a fluorinated pyrimidine antimetabolite. It inhibits thymidylate synthetase, which ultimately interferes with DNA, and to a lesser degree, RNA synthesis. Fluorouracil appears to preferentially affect the G1 and S phases of the cell cycle.

Pathophysiology of PPE

As previously described, the conversion of capecitabine to 5-FU requires a series of enzymatic reactions. Genetic polymorphisms may play a role in increasing a person’s susceptibility to exhibiting signs and symptoms of toxicity. Although there are about 25 candidate genes identified in which variation may affect the efficacy and toxicity of capecitabine therapy, two specific genes are most commonly mentioned. Polymorphisms involving the genes that encode thymidylate synthase (TYMS) and dihydropyrimidine dehydrogenase (DPD) have been mentioned as possibly contributing to the development of toxicity with capecitabine therapy. While genetic tests do exist to detect such polymorphisms, it is not standard of care to conduct testing prior to initiating fluoropyrimidine therapy.

Of all of the genetic polymorphisms thought to be related to fluoropyrimidine intolerance, the gene that encodes DPD has been most studied. About 3-5% of Caucasians have a partial DPD deficiency, and 0.2% have a complete deficiency. All patients who are found to have less than 70% DPD activity are considered to be at risk for the development of severe drug-related toxicities [10]. In the literature review, no case of PPE with genital involvement mentioned testing for genetic polymorphisms, except for one. The one patient that was tested was found to have no mutation [5].

The pathophysiology of PPE is not well understood. Those without a genetic polymorphism still can experience signs and symptoms of toxicity, specifically PPE. One proposed mechanism is trauma to the palms and soles from normal daily activities leading to the rupture of small dermal capillaries. This allows the release of the drug into the surrounding tissues, causing an inflammatory response. The scrotum and genitalia could possibly be considered to sustain a fair amount of trauma during daily activities, but not nearly to the extent of the palms of the hands and soles of the feet [11].

Another mechanism that has been described focuses on the eccrine sweat glands. The palms, soles, scalp, and genitals contain the highest concentrations of eccrine sweat glands on the body. There are two different theories that involve the eccrine sweat glands.

One theory is that noninflammatory metaplasia of cuboidal epithelial cells of the eccrine sweat ducts occurs as a result of the direct toxic effects of the drug, known as eccrine squamous syringometaplasia (ESS). ESS appears from two to 39 days after initiation of chemotherapy and is reported as resolving spontaneously in 7-10 days. It has been suggested that histologic analysis may assist in differentiating ESS from other possible diagnoses [12]. 

A second theory that involves the eccrine sweat glands describes the accumulation of the drug on the skin surface, allowing it to penetrate the upper epidermis. Here, it shows cumulative toxic effects by forming free radicals that damage cellular structures. The same mechanism was implicated in the cutaneous signs and symptoms associated with pegylated liposomal doxorubicin therapy, and subsequently successfully targeted in preventing and treating the condition.

Treatment and prevention of PPE

Various treatments for PPE have been suggested, all with varying degrees of efficacy. Ultimately, what is required in most cases is either interruption or discontinuation of the treatment. Out of eight cases of PPE with genito-scrotal involvement reported in the literature, including this case, seven required discontinuation of chemotherapy.

As with the appearance of any sign or symptom of toxicity, dose reduction may be a reasonable course of action. Lengthening the intervals of the drug administration schedule alone, or in addition to dose reduction, may be effective [1, 13].

Prophylactic measures may be taken in an attempt to prevent PPE from occurring. One systemic medication that has been frequently discussed is celecoxib. Celecoxib is a cyclooxygenase-2 (COX-2) inhibitor. Because some of the theories about the pathophysiology of PPE revolve around a local inflammatory tissue reaction after extravasation of the drug allowing it to penetrate the stratum corneum likely mediated by COX-2, it was thought that treatment with celecoxib may decrease the likelihood of development of PPE. In a meta-analysis, celecoxib was found to be associated with a statistically significant reduction of moderate to severe PPE. The authors claim that it is the “most promising” agent for the prevention of PPE. Some of the well-known potential side effects of celecoxib, however, have led some to not be in support of its use for this purpose [14].

Pyridoxine (vitamin B6) is an agent that is commonly administered to patients undergoing chemotherapy, with the intention of preventing PPE. It has been suggested that the use of pyridoxine to either prevent or treat PPE is based on poor evidence. The use of pyridoxine arose when similarities between PPE and acrodynia from pyridoxine deficiency was noted in rats. Two separate studies, one randomized clinical trial, and one meta-analysis both came to the same conclusion [14, 15]. There is no clinical evidence to support the use of pyridoxine to prevent or ameliorate PPE.

Topical urea is a cream that is applied to the hands and feet. At low concentrations, it acts to retain moisture. This occurs because topically, urea promotes the uptake of water by the stratum corneum by allowing it to have a high water-binding capacity. In a meta-analysis, urea cream failed to demonstrate an advantage in the prevention of all-grades of PPE (Table 1) [16].

**Table 1:** PPE common terminology of adverse events Adopted from [9]. PPE - palmar-plantar erythrodysesthesia; ADL - activities of daily living

Grade	Explanation
Grade 1	Minimal skin changes or dermatitis (e.g., erythema, edema, or hyperkeratosis) without pain
Grade 2	Skin changes (e.g., peeling, blisters, bleeding, edema, or hyperkeratosis) with pain; limiting ADL
Grade 3	Severe skin changes (e.g., peeling, blisters, bleeding, edema, or hyperkeratosis) with pain; limiting self-care ADL

Systemic and topical steroids have been used to prevent and/or treat PPE. Their use has been poorly studied, especially in regard to capecitabine-associated PPE. Most of the literature regarding the use of steroids to treat and/or prevent PPE is in relation to pegylated liposomal doxorubicin. Even then, the literature is quite sparse. A study by Drake et al. used dexamethasone to treat those who experienced PPE after receiving pegylated liposomal doxorubicin for various gynecologic malignancies. In that study, nine patients developed PPE. Six received a dexamethasone taper. All six demonstrated a complete or near-complete resolution of PPE and required no dose modification. All three who did not receive steroids required treatment delays and dose reductions [16].

Application of topical antiperspirant has been hypothesized to be a potentially beneficial measure in preventing PPE given the idea that accumulation of the drug on the surface of the skin may occur via the eccrine sweat glands. One study suggests that the application of topical aluminum chlorohydrate is well-tolerated and may reduce the incidence of grade 2 or 3 PPE (Table 1) [17].

With fairly new technology allowing for the detection and measurement of concentrations of capecitabine on the surface of the skin, some parallels may be drawn between pegylated liposomal doxorubicin-associated PPE and capecitabine-associated PPE. Pegylated liposomal doxorubicin spreads evenly on the skin surface, penetrating into the superficial epidermis. There it shows cumulative toxic effect by forming free radicals that injure cellular structures. The topical application of an antioxidant ointment was found to be effective in preventing and treating PPE [18]. This theory was tested in patients who developed PPE secondary to capecitabine. Topical Mapisal®, an ointment that contains several antioxidants and has a high radical protection factor, was studied in comparison to topical 10% urea cream. The urea cream was found to be superior [19]. The topical application of silymarin, another agent rich in antioxidants, has been studied and found to potentially be useful in delaying the onset of PPE [19]. Given that one of the theories regarding the etiology of PPE is the accumulation of the drug on the skin surface due to sweating, it would be reasonable to attempt to mitigate the effects by controlling sweating. Regional cooling has been suggested as an intervention to prevent PPE. One study described placing ice packs around the wrists and ankles of patients receiving pegylated liposomal doxorubicin therapy for gynecologic malignancies. The authors indicated that the study demonstrated the intervention was an efficacious prevention strategy [20].

## Conclusions

PPE is a common adverse reaction to capecitabine, as well as many other drugs used to treat malignancies. Involvement of the genitals is rare but seems to be associated with increased severity of the adverse reaction. Although genital involvement has been reported with a few other drugs, it could potentially occur with the administration of any drug associated with the development of PPE. The symptoms can be debilitating, so early detection is prudent. This may mean that patients receiving capecitabine therapy need to be specifically asked about skin changes or pain in the genitals, or even have regular genital examinations.
